# Synthesis of Iron on Carbon Foam for Use in the Removal of Phenol from Aqueous Solutions

**DOI:** 10.3390/molecules28031272

**Published:** 2023-01-28

**Authors:** Siphesihle Praise-God Khumalo, David Lokhat, Chante Jasmine-Tre Anwar, Huvin Reddy

**Affiliations:** School of Engineering, Discipline of Chemical Engineering, University of KwaZulu-Natal, Durban 4000, South Africa

**Keywords:** adsorption, phenol, carbon foam

## Abstract

The potential use of magnetic nanopowder for phenol adsorption mobilised on natural grain carbon foam from an aqueous solution was studied. Phenolic compounds are priority pollutants with high toxicity even at low concentrations. A magnetic nanopowder was synthesised by dissolving an iron sponge in nitric acid to produce iron nitrate, which was added to a natural grain mixture with flour as the main ingredient. The synthesised carbon foam was investigated for the effects of initial concentration, time, and TEM (transmission electron microscopy) characterisation. The phenol adsorption increased as the iron content of the carbon foam and the initial concentration increased. A kinetic study showed that the phenol adsorption data adequately covered all the carbon foam samples tested using an equation corresponding to a pseudo-first order chemical reaction. The Freundlich, Langmuir, and Temkin equations were tested for modelling the adsorption isotherms at equilibrium, and it was concluded that the Temkin model fit the experimental data adequately. Due to its exceptional physical and chemical properties, carbon magnetic nanopowder is regarded as an outstanding pollutant absorber in environmental investigations. R^2^ values derived from the pseudo-first-order model exceed 0.99. R^2^ > 0.94 indicates that the Freundlich isotherm provides the best fit to the equilibrium data.

## 1. Introduction

In recent years, the removal of phenol has become recognised as an important area of research within the discipline of chemical engineering [1]. Phenolic compounds are high on the priority pollutants list because they can pose a significant risk even in small amounts [2]. Phenol that is contained in wastewater can form complicated compounds by combining with the metal ions that are discharged from other industries [3]. Phenols are used to manufacture phenolic resins, epoxy resins, adhesives, and polyamide for a variety of commercial applications. Coal conversion at high temperatures, petroleum refining, resins, and plastics all produce wastewater containing phenolic pollutants. Aromatic hydroxy compounds, which are toxic at high concentrations and suspected carcinogens, are a top pollutant. Therefore, phenol must be removed from industrial effluents prior to their discharge into waterways [4]. Even at low concentrations, phenolic chemicals are harmful. If these compounds are released without first being handled, it is probable that significant health problems may be posed to both human and animal populations as well as to aquatic ecosystems [5]. The establishment of rigorous discharge regulations for phenols has been mandated by international regulatory organisations to make certain that the environment will remain habitable [6]. 

To properly extract phenolic compounds from wastewater before it is disposed of in water resources, a variety of technologies are used [7]. Typical removal methods include steam distillation, liquid–liquid extraction, adsorption, solid-phase extraction, wet air oxidation, catalytic wet air oxidation, and biodegradation [8]. Electrochemical oxidation, photo-oxidation, ozonation, UV/H_2_O_2_, the Fenton reaction, membrane processes, and enzymatic treatment are among the most advanced methods for removing phenols [9]. Physical, chemical, and biological treatment are the three conventional categories for the elimination of phenolic contaminants from aqueous solutions [10]. 

Adsorption is the most efficient method for removing organic and inorganic contaminants from wastewater [11] because it is simple to set up, inexpensive, does not require much time, and the adsorbent used in the process is not harmful to the environment and can be recovered and reused without a reduction in efficiency [12]. Therefore, the hunt for inexpensive and readily accessible adsorbents has prompted numerous researchers to seek more cost-effective and effective strategies for employing natural and synthetic materials as adsorbents [13]. The most extensively employed adsorbent is activated carbon. It has a high capacity for the adsorption of organic molecules [14].

Carbon foam’s properties, such as its specific surface area, surface functional groups, pore size distribution, and other surface characteristics, may affect its ability to absorb phenol and how it does so.

The primary advantages of carbon as an adsorbent over other techniques are its ability to remove both organic and inorganic chemicals by batch or column methods and its regeneration after repeated usage. Recent research, however, has proven that nanoparticles have highly specific surface areas [15].

Agriculture wastes such as coir pith, waste wood, orange peel, bagasse, coffee husk, pinecone, sunflower seed hull, coconut tree, hazelnut husks, rice hulls, oil palm shell, coconut husk, and pine-fruit shell were effectively converted into activated carbon. Carbonisation is the process of removing non-carbon components, such as oxygen and hydrogen, in gaseous form by pyrolysis. However, the adsorption capacity of the resulting carbonised product is still limited, necessitating the immobilisation of nanoparticles to increase its adsorption [7]. Even though various research has been published on the use of metal nanoparticles as catalysts, this article focuses on the application as a sorbent, which is less demanding and may be robustly made using low-cost materials.

Magnetic nanopowder is thought to be one of the most effective and cost-efficient adsorbents for removing organic and inorganic pollutants from aquatic environments [16]. It has a high surface area and a high porosity, and it is cheap, abundant, made from renewable sources, stable at high temperatures, and resistant to all chemicals [1]. Hence, in this study, the use of magnetic nanopowder for phenol adsorption on natural grain carbon foam to reduce the concentration of phenol in diverse solutions was investigated. In addition to altering the phenol content in the solution while maintaining the nanoparticle mass, stirring time, and stirring speed constant, additional studies were conducted to determine the impact of nanoparticle contact time with the phenol solution. 

In this study, the carbon precursor and magnetic nanopowder precursor were mixed and carbonised in a single vessel. When the natural grain is baked and carbonised at the same time, a porous structure is formed that can support the iron oxide particles well.

## 2. Results and Discussion

### 2.1. Characterisation of Carbon Foam

**Figure 1:**
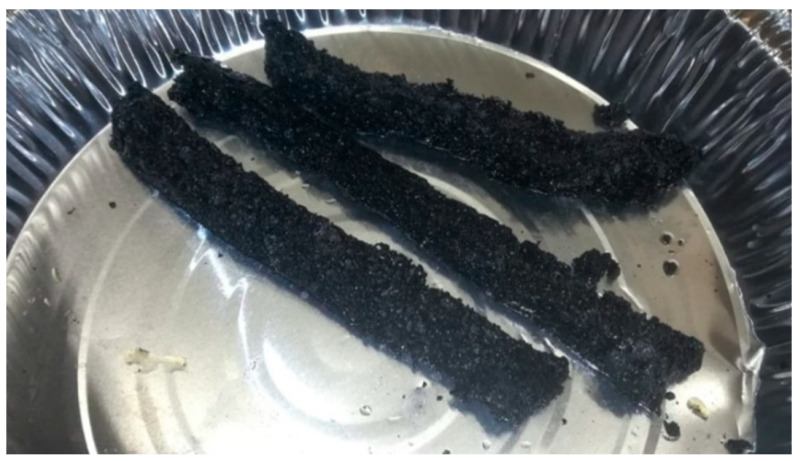
Photograph of carbon foam from the furnace.

**Figure 2:**
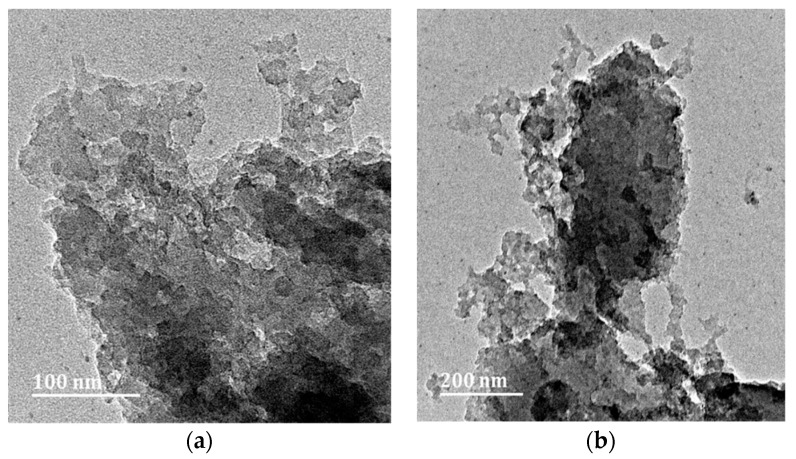

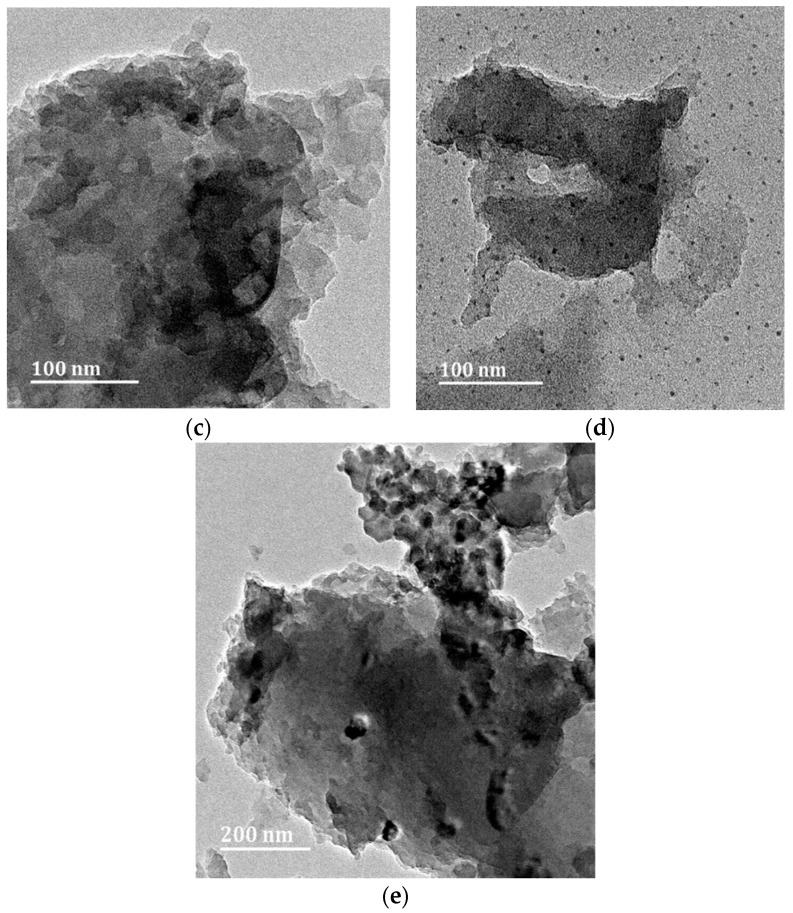
TEM image conducted on a Jeol 2100 HRTEM operating at 200 KV: (**a**) Sample 1, (**b**) Sample 2, (**c**) Sample 3, (**d**) Sample 4, and (**e**) Sample 5.

The presence of iron accelerates the action of yeast, namely, the consumption of sugars in the grain combination and the creation of carbon dioxide, which is responsible for the formation of pores in the carbonised material. The presence of iron nanoparticles enhances the adsorption capabilities of an adsorbent. Due to their ultra-fine diameters and increased surface areas, these iron oxide nanoparticles have demonstrated a remarkable capacity for the adsorption of pollutants with high reactivity, high removal efficiencies, and quick operation [17]. Phenol adsorption to negatively charged iron nanoparticles is predominantly promoted by electrostatic interactions and is very sensitive to pH and ionic strength changes. Anionic (negatively charged) iron oxide nanoparticles dispersed in deionised water and cationic phenol, which adsorbs onto the nanoparticles’ surface and initiates their aggregation. In the presence of a magnetic field, dipolar interactions between primordial agglomerates induce agglomeration [18]. The existence of cavities in the carbon structure is advantageous for the adsorption process because they allow phenol molecules to penetrate the adsorbent. Figure 1 shows carbon foam samples after carbonisation from the furnace. Figure 2a–e depicts the TEM micrographs of the iron immobilised in natural grain carbon foam. It is evident that the addition of iron nitrate particles to carbon foam produced a structure that is more amorphous and porous, see Figure 2. The surface of the material featured geometrical properties, such as an irregular form, large agglomerates, and a rough surface, which would provide extra sites for adsorbing heavy metal ions [19]. Figure 2a,b,d illustrate the findings of a TEM investigation at 200 nm magnification, which demonstrated that iron oxide was successfully distributed across the surface of the carbon foam. The presence of iron oxide is indicated by the number of dark crystallites in the image. If the iron concentration in the sample is too high, as depicted in Figure 2c,e, agglomeration may occur, which enlarges the iron particles and reduces the sorbent’s surface area and adsorptive properties [20]. These characteristics have been demonstrated to be helpful for synthetic carbon foam.

### 2.2. Effect of Initial Concentration

**Figure 3:**
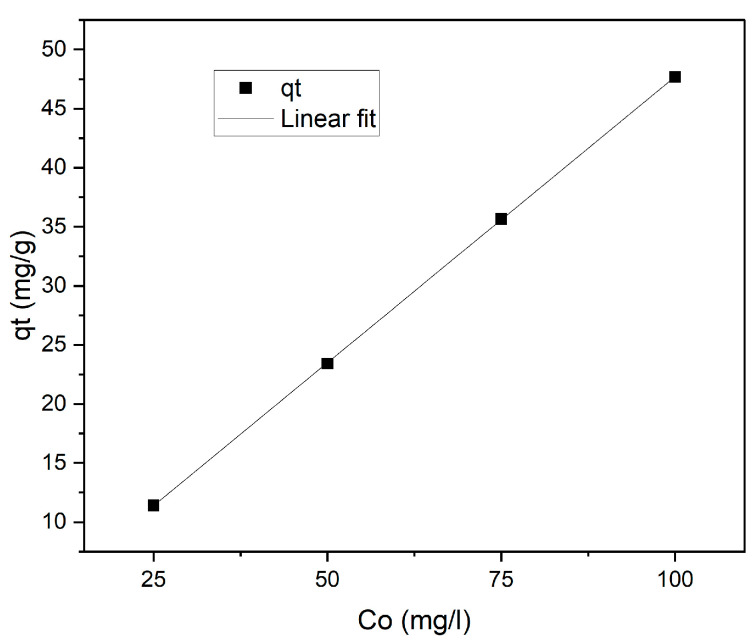
The effect of initial concentration on adsorption.

Shaking the adsorbent–adsorbate solution until equilibrium is reached for increments of the initial adsorbate concentration at a similar adsorbent dosage and time can be used to study the effect of the initial concentration [21]. Adsorption depends on the initial concentration of an adsorbate, since lowering the initial concentration of phenol molecules lowers the surface area. As the initial concentration increases, the driving force of mass transfer increases, enhancing adsorption [22]. Figure 3 shows the effect of the initial concentration of phenol, where initial concentrations of 25, 50, 75, and 100 mg/L were adsorbed by iron immobilised on natural grain carbon foam in four different samples. There was an increase in adsorption with an increase in the initial concentration at room temperature with a contact time of 10 min and 25 mg/L of solution. The amount of available surface area on adsorbents goes down as the initial concentration increases. This means that more adsorbate is taken up and less phenol is removed.

The following equation was used to calculate the amount of phenol adsorbed [19]:

(1) $$q_{t}=\frac{\left(C_{0}-C\right)\times v}{w}$$

where *q_t_* (mg/g) is the adsorption density, *C*_0_ (mg/L) is the initial concentration of aqueous solution, *C* (mg/L) denotes the final aqueous solution concentration (mg/L), *v* (L) the volume of solution, and *w* (g) the mass of adsorbent.

**Figure 4:**
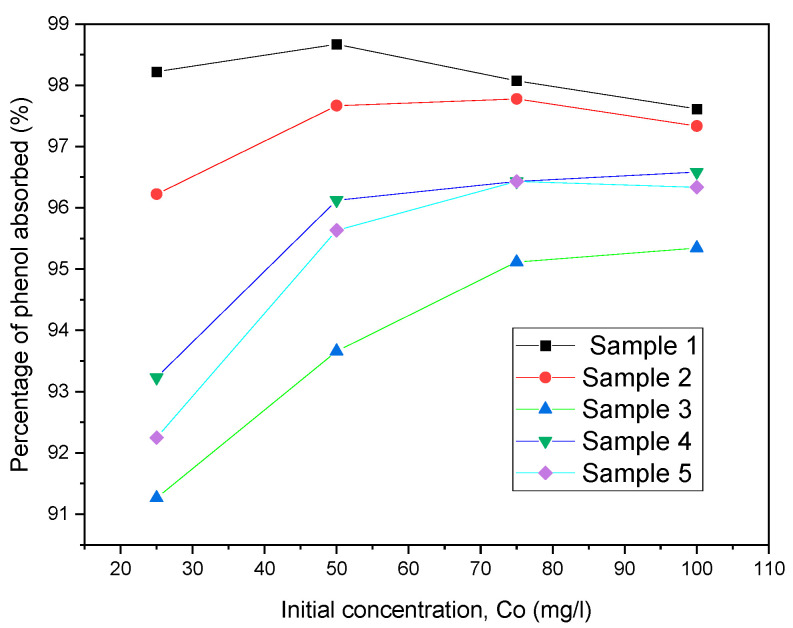
The effect of initial concentration on percentage of phenol adsorbed (50 mL of phenol solution and 0.1 magnetic nanopowder).

Four phenol solutions were prepared with concentrations ranging from 25, 50, 75, and 100 mg/L. A plot of the percentage of phenol adsorbed versus the initial concentration was made for each sample of nanopowder in each solution concentration. Figure 4 indicates that the percentage of phenol adsorbed increased for each sample as the initial concentration increased. This demonstrates that more light is being absorbed by a solution of greater concentration. This makes sense because when the concentration of a solution goes up, there are more particles for the light to hit, so there are more chances for absorption [23].

### 2.3. The Effect of Time on Adsorption

Adsorbate rate rises with longer contact times because it deposits on the accessible adsorption sites on the adsorbent material; nevertheless, longer contact times will not result in an increase in uptake [24]. The amount of adsorbate adsorbed onto the adsorbent is in equilibrium with the amount of adsorbate desorbing from the adsorbent. The effectiveness of adsorbate removal is substantially impacted by the length of time that the two components are in contact [3].

**Figure 5:**
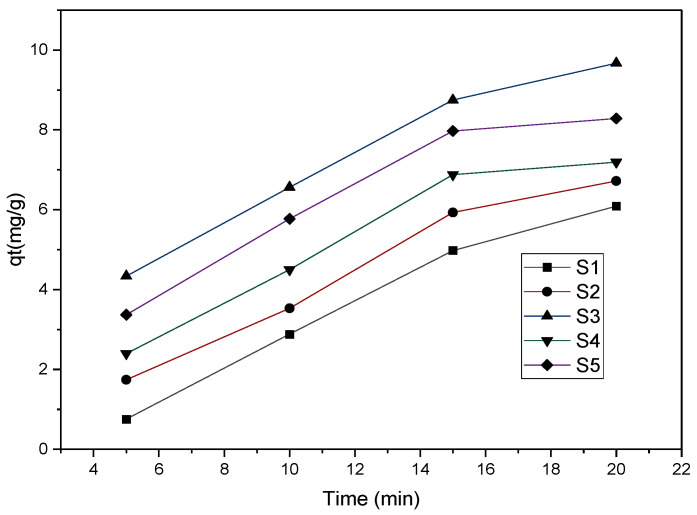
The effect of contact time on the adsorption of phenol at concentration of 75 mg/L and 100 mg.

Figure 5 displays the results obtained, and for each sample, phenol adsorption increased with contact time. The magnetite solution displayed the least phenol adsorption. This can be attributed to the uneven distribution of the solid particles in the initial organic mixture. The sample of undissolved iron sponges yielded similar results to the previous sample for the same reasons. The three samples that made use of liquid iron nitrate yielded better results, as there was more even distribution in the organic mixture, resulting in a sample of constant composition. An increase in the amount of iron sponge added to the mixture increased phenol adsorption. A clarification for a decline in the adsorption rate after 15 min is that the solutions are approaching equilibrium. This can be attributed to the large number of vacant sites on the magnetic nanoparticle surface, which are gradually occupied over time as adsorption takes place.

### 2.4. Mechanism

Using kinetic models, the rate of the adsorption process and the rate-controlling step are explored to fully comprehend the phenomena involved in phenol adsorption on magnetic nanopowder. In addition, it is crucial to consider the fact that the adsorption mechanism is dependent on the physical and/or chemical properties of the adsorbent as well as the mass transport process. To investigate the mechanism of phenol adsorption onto magnetic nanopowder, pseudo-first-order, pseudo-second order, and intraparticle diffusion models are considered to fit the experimental data obtained from batch studies, and the correlation coefficient is regarded as a measure of the relationship between the experimental data and these proposed models.

Since intraparticle diffusion was not the only rate-limiting step in the adsorption of phenol onto iron supported by carbon foam, the adsorption rate was controlled by a multistep elementary reaction mechanism. Other processes may also affect the adsorption rate, and they may all be active concurrently. Consequently, the Temkin isotherm best characterises the phenol adsorption process, which suggests multilayer adsorption over a heterogeneous surface with varied energy distribution. On the adsorption isotherm, the Temkin model reflects the features of indirect adsorbate–adsorbent interactions. Due to adsorbate–adsorbent interactions, it is believed that the heat of adsorption of all molecules in the layer reduces linearly with coverage. Due to adsorbate–adsorbent interactions, the adsorption of all molecules in the layer reduces linearly with increasing layer coverage. Phenol adsorption on negatively charged iron nanoparticles is primarily facilitated by electrostatic interactions and is extremely sensitive to changes in pH and ionic strength. Nanoparticles of anionic (negatively charged) iron oxide are distributed in deionised water with cationic phenol, which adsorbs on the nanoparticles’ surface and triggers their aggregation. In the presence of a magnetic field, primordial agglomerates form due to dipolar interactions.

The mechanism for phenol adsorption on carbon foam as an adsorbent is based on their mainly sp2-dominated electronic structures. As a result, one could speculate that the adsorption mechanism for this system involves interactions between delocalised electrons on the carbon surface structure and electrons from the aromatic ring [25].

### 2.5. Kinetic Modelling

Lagergren pseudo-first order and pseudo-second order models were used for the investigation of modelling of adsorption kinetics. The nonlinear form of pseudo-first order is given by the following equation:

(2) $$q_{t}=q_{e}\left(1-e^{-K_{1}t}\right)$$

**Figure 6:**
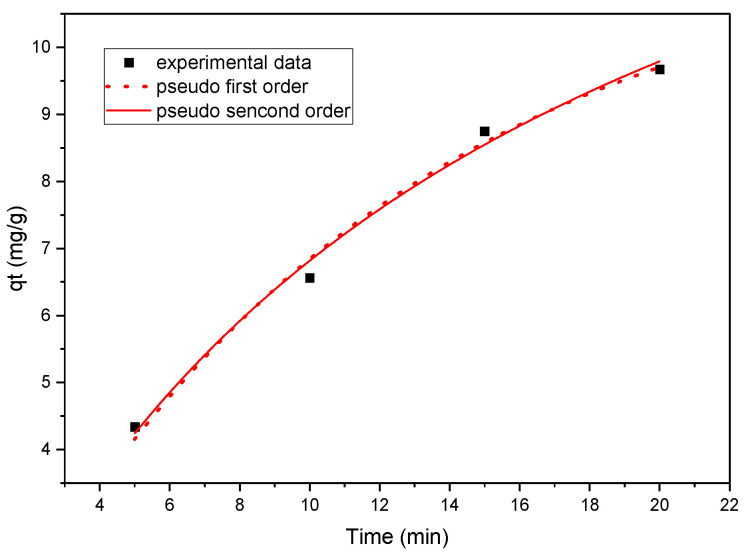
Nonlinear fit of pseudo-first order and pseudo-second order for phenol adsorption of sample 3 at a 75 mg/L of concentration and 100 mg sorbent.

The nonlinear form of pseudo-second order is given by the following equation:

(3) $$q_{t}=\frac{q_{e}{}^{2}K_{2}t}{1+K_{2}q_{e}t}$$

where *q_t_* (mg/g) is the amount absorbed at time *t*, *q_e_* (mg/g) is the amount remaining after equilibrium of adsorption, and *K*_1_ and *K*_2_ are the pseudo-first and pseudo-second order model rate constants, expressed in min^−1^ and g/mg/min, respectively. Table 1 and Table 2 illustrates the calculated values of *q_e_*, *K*_1_, and *K*_2_, and the regression coefficient R^2^ values. Figure 6 shows the plot of the pseudo-first order and pseudo-send order. The validity of the model is judged by evaluating the correlation coefficients R^2^ and the comparability of the experimental and calculated values of *q_e_*. Considering these findings, it is possible to conclude that the pseudo-first-order kinetic model gives a stronger correlation for phenol adsorption onto magnetic nanopowder immobilised on carbon foam than the other models. The results indicate that R^2^ is closer to 1 for pseudo-first order compared to the pseudo-second order model for all five samples.

### 2.6. Intraparticle Diffusion 

**Figure 7:**
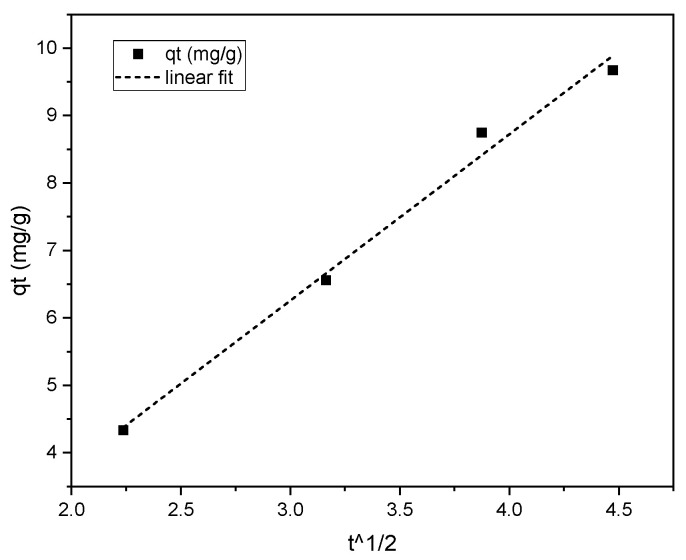
Plot of the intraparticle diffusion model for phenol onto iron supported by carbon foam (75 mg/L of concentration, and 100 mg sorbent).

The intraparticle diffusion resistance was evaluated utilising the intraparticle particle diffusion model by Weber and Morris [26], given by the following equation:

(4) $$q_{t}=k_{id}t^{1/2}+c$$

where *q_t_* (mg/g) is the adsorption amount at time *t* (min), *k_id_* (mg/g/min^1/2^) is the adsorption rate constant of the intraparticle diffusion model, and c is a constant related to the thickness of the boundary layer. The plot illustrated in Figure 7 is linear, and the sorption process is solely regulated by intra-particle diffusion since the plot of *q_t_* versus *t*^1/2^ yields a straight line. The difference in mass transfer rates between the start and final stages of adsorption may be the cause of the plot’s departure from the origin. If intraparticle diffusion is involved in the sorption process, a graph of adsorbate uptake vs. the square root of time would have a linear connection, and intraparticle diffusion would be the rate-regulating step if this line passes through the origin [27].

Furthermore, such a departure from the origin of the straight line suggests that pore diffusion is not the only factor affecting rate [28].

### 2.7. Equilibrium Adsorption

Adsorption equilibrium occurs between the adsorbed molecules and the adsorbent surface when an adsorbate is in contact with the adsorbent. The adsorption isotherm is the equilibrium relationship between the amount of adsorbed adsorbate (*q_e_*) and the residual adsorbate concentration (*C_e_*) at a constant temperature. In general, adsorption isotherms provide information on the affinity and the binding energy between the adsorbate and the adsorbent, on the adsorption capacity, and on the surface phase, which may be considered a monolayer or multilayer. The modelling of the adsorption isotherms consists of describing the exponential data using theoretical or empirical mathematical equations and allowing the determination of isotherm parameters to compare the efficiency of different adsorbents. 

Three adsorption equilibrium isotherm models were tested in the present research, namely, the Freundlich, Langmuir, and Temkin isotherm models. The empirical Freundlich model, which is known to be satisfactory for low concentrations and is based on sorption on the heterogeneous surface, is expressed by the following equation:

(5) qe=KFCe1n

where *C_e_* (mg/L) is the equilibrium concentration of adsorbate, *q_e_* (mg/g) is the amount of adsorption at the equilibrium. *K_F_* and *n* are Freundlich constants related to the adsorption capacity and adsorption intensity, respectively. All the parameters of the isotherm models were calculated from the nonlinear fitting of *q_e_* versus *C_e_* on Origin Lab software. The Temkin model reflects the properties of indirect adsorbate–adsorbent interactions on the adsorption isotherm. It is assumed that the heat of adsorption of all molecules in the layer decreases linearly with coverage due to adsorbate–adsorbent interactions. The Temkin model is expressed by the following equation:

(6) $$q_{e}=\frac{RT}{b}ln\left(AC_{e}\right)$$

where *q_e_* (mg/g) is the amount of adsorption at the equilibrium, *C_e_* (mg/L) is the equilibrium concentration of adsorbate, *T* (K) is the temperature in Kelvin, *R* (J/mol/K) is the universal gas constant, *b* (J/mol) is related to the heat of adsorption and *K_T_* is the equilibrium binding constant corresponding to the maximum binding energy. 

The Langmuir isotherm equation is expressed as follows:

(7) $$q_{e}=\frac{q_{m}K_{L}C_{e}}{1\pm K_{L}C_{e}}$$

where *q_e_* (mg/m) is the monolayer adsorption capacity, *C_e_* (mg/L) is the equilibrium concentration of adsorbate, *q_m_* (mg/g) is the monolayer adsorption capacity, and *K_L_* is the Langmuir constants.

**Figure 8:**
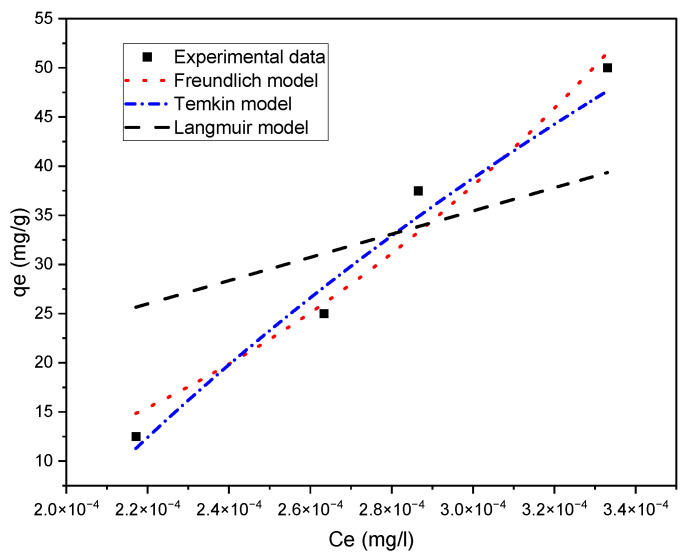
Fitting of adsorption isotherms into experimental data for sample 3.

Figure 8 illustrates the curves of the Freundlich, Temkin and Langmuir models using Equations (5)–(7), respectively. Table 3 depicts the calculated parameters of the Freundlich and Temkin isotherms, the R^2^ values obtained by the nonlinear fitting method. Based on the R^2^ value comparisons, the Freundlich model represents a better fit of the experimental data at equilibrium compared to both the Langmuir and Temkin models. Thus, the Temkin isotherm best describes the phenol adsorption process, which indicates multilayer adsorption on a heterogeneous surface with different energy distribution. The Freundlich constant, n, is a measure of adsorption intensity. A value of 1/n was found to be between 0 and 1, indicating that Freundlich was also favourable for the adsorption of phenol [29].

## 3. Materials and Methods

### 3.1. Materials and Chemicals

The following materials were used: deionised water, phenol, iron oxide nanoparticles, iron sponge, nitric acid, magnetite, flour, and yeast.

### 3.2. Preparation of Carbon Foam

Phenol removal is a topic of particular interest in the field of preventing and getting rid of pollution because it is toxic to water quality and can hurt people even in small amounts. Adsorption is still one of the best ways to get rid of something. It became possible when magnetic nanopowder was made in a carbon foam suspension. The 5 grammes of dry yeast was dissolved in 115 mL of water with continuous stirring. Once the yeast and water were completely dissolved, the mixture was added to 300 g of flour. A varying amount of iron nitrate was added into the flour–yeast mixture. This was performed by mixing iron sponge with nitric acid to make iron nitrate, which was then added to a mixture of natural grains to bake. The mixture was then mixed with water, and the mechanical mixer was used to knead the mixture until it formed a paste. The porous structure was made by letting the paste ferment in an oven at 35 °C for an hour. The bread was then allowed to bake in an oven set at 180 °C for 40 min. Thereafter, the bread was allowed to dry in an oven at 80 °C for 18 h. A U-tube furnace under argon gas conditions was set up. The dry bread was placed into the furnace and allowed to carbonise at 10 °C/min. The oven was set to heat up at a rate of 10 °C per minute and reach a maximum temperature of 600 °C. The holding time for the maximum temperature was set at 120 min. The sample was allowed to cool in the argon atmosphere until it reached room temperature. When different amounts of liquid iron nitrate were added to the flour, yeast, and water mix, four different organic samples were made. To make a second sample, magnetite was added to the dry ingredients, and then water was added. Table 4 depicts the iron contents for each sample.

### 3.3. Adsorption of Phenol

The beaker-based adsorption studies were conducted in batch model, where 0.1 g of iron oxide nanoparticles was added to 50 mL of the 25 mg/L solution, and the resulting mixture was agitated with an overhead stirrer at 500 rpm for 10 min. Filtration was then used to remove the nanoparticles from the solution, and the clear filtrate was placed into the photocell for testing. This digital absorbance and transmittance data were collected so that the solution’s ultimate concentration could be determined using the calibration curve. The stages were performed for each concentration of phenol and water solution at room temperature, roughly 25 °C.

## 4. Conclusions

The amount of phenol adsorbed for these tests all increased with the increase in initial concentration. Adsorption also increased with the increase in iron content in the carbon form, and the magnetite sample had lower adsorption capabilities compared to other samples. The adsorption values increased rapidly with time on the *q_t_* (mg/g) versus time (min) graph, and then they started to level out as the solutions got closer to equilibrium. This was attributed to the fact that there were a lot of open sites on the magnetic nanoparticle surface, which over time, as adsorption occurred, gradually filled up. This study demonstrates that magnetic nanopowder immobilised on carbon foam significantly reduces phenol concentrations. The correlation between the adsorption data and the pseudo-first-order equation is the highest. The Temkin adsorption model is more suitable for describing the phenol adsorption equilibrium data.

## Figures and Tables

**Table 1:** Pseudo-first order parameters.

	Exp. qe	Calc. qe	K_1_	R^2^
Sample 1		39.99 × 10^2^	7.64 × 10^−8^	0.953
Sample 2	7.3	26.21	0.015	0.974
Sample 3	6.5	11.76	0.872	0.992
Sample 4	9.8	11.84	0.050	0.963
Sample 5	8.5	10.74	0.080	0.976

**Table 2:** Pseudo-second order parameters.

	Exp. qe	Calc. qe	K_2_	R^2^
Sample 1		46.54 × 10^2^	1.42 × 10^−8^	0.95
Sample 2	7.3	50.49	1.58 × 10^−4^	0.97
Sample 3	6.5	17.22	3.79 × 10^−4^	0.99
Sample 4	9.8	19.88	1.54 × 10^−3^	0.96
Sample 5	8.5	16.34	3.41 × 10^−3^	0.97

**Table 3:** Temkin, Freundlich, and Langmuir model parameters.

Sample	Temkin			Freundlich			Langmuir		
	b	K_T_	R^2^	n	K_F_	R^2^	q_m_	K_L_	R^2^
1	38.76	7920.14	0.98	1.97	16,564.30	0.91	82750.9	1.96	0.78
2	30.89	5480.35	0.94	2.35	60,792.30	0.87	88,020.60	1.19	0.66
3	29.16	5260.43	0.98	2.91	401,434.60	0.97	105,489.00	1.12	0.61
4	24.53	4494.55	0.97	2.99	577,825.00	0.93	159,254.50	0.67	0.52
5	23.1	4075.37	0.94	2.92	252,312.60	0.87	83,231.50	1.27	0.55

**Table 4:** Iron and magnetite added into carbon form for each sample.

Sample 1	Sample 2	Sample 3	Sample 4	Sample 5
Magnetite	3 g pure iron	3 g iron sponge dissolved in nitric acid	1 g iron sponge dissolved in nitric acid	2 g of iron sponge dissolved in nitric acid

## Data Availability

Not applicable.

## Sample Availability

Samples of the compounds are available from the authors.

## References

[1] Fathy Mubarak M., Maher Ahmed A., Saad Gabr S. (2021). Nanoporous carbon materials toward phenolic compounds adsorption. Nanopores.

[2] Bagheri B., Hosseini S.A., Mehrizadeh H. (2020). A Comparison of the Catalytic Activity of Cu-X_2_ (X = Mn, Co) Nano Mixed Oxides toward Phenol Remediation from Wastewater by Catalytic Wet Peroxide Oxidation. J. Water Environ. Nanotechnol..

[3] Fierro V., Torné-Fernández V., Montané D., Celzard A. (2007). Adsorption of phenol onto activated carbons having different textural and surface properties. Microporous Mesoporous Mater..

[4] Kandisa R.V., Saibaba K.N., Shaik K.B., Gopinath R. (2016). Dye Removal by Adsorption: A Review. J. Bioremediat. Biodegrad..

[5] Adeleye A.S., Conway J.R., Garner K., Huang Y., Su Y., Keller A.A. (2016). Engineered nanomaterials for water treatment and remediation: Costs, benefits, and applicability. J. Chem. Eng..

[6] Villegas LG C., Mashhadi N., Chen M., Mukherjee D., Taylor K.E., Biswas N. (2016). A Short Review of Techniques for Phenol Removal from Wastewater. Curr. Pollut. Rep..

[7] Asencios Y.J.O., Lourenço V.S., Carvalho W.A. (2022). Removal of phenol in seawater by heterogeneous photocatalysis using activated carbon materials modified with TiO_2_. Catalysis Today.

[8] Pirillo S., Pedroni V., Rueda E., Ferreira M.L. (2009). Elimination of dyes from aqueous solutions using iron oxides and chitosan as adsorbents: A comparative study. Química Nova.

[9] Mohammadi S., Kargari A., Sanaeepur H., Abbassian K., Najafi A., Mofarrah E. (2015). Phenol removal from industrial wastewaters: A short review. Desalination Water Treat..

[10] Oyelude E.O., Owusu U.R. (2011). Adsorption of Methylene Blue from Aqueous Solution Using Acid Modified Calotropis Procera Leaf Powder. J. Appl. Sci. Environ. Sanit..

[11] Shahid M., Farooqi Z.H., Begum R., Arif M., Irfan A., Azam M. (2020). Extraction of cobalt ions from aqueous solution by microgels for in-situ fabrication of cobalt nanoparticles to degrade toxic dyes: A two fold-environmental application. Chem. Phys. Lett..

[12] Turco A., Monteduro A.G., Mazzotta E., Maruccio G., Malitesta C. (2018). An Innovative Porous Nanocomposite Material for the Removal of Phenolic Compounds from Aqueous Solutions. Nanomaterials.

[13] Gupta S., Babu B.V. (2010). Experimental, kinetic, equilibrium and regeneration studies for adsorption of Cr(VI) from aqueous solutions using low cost adsorbent (activated flyash). Desalination Water Treat..

[14] Ho Y.S., McKay G. (1998). Kinetic models for the sorption of dye from aqueous solution by wood. Process. Saf. Environ. Prot..

[15] Arif M. (2022). Complete life of cobalt nanoparticles loaded into cross-linked organic polymers: A review. R. Soc. Chem..

[16] Negrescu A.M., Killian M.S., Raghu S.N., Schmuki P., Mazare A., Cimpean A. (2015). Metal Oxide Nano-particles as an Adsorbent for Removal of Heavy Metals. J. Adv. Chem. Eng..

[17] Arif M., Munawar K., Ali S., Raza H., Ashfaq M. (2020). Synthesis and Optical Study of Sensitive and Selective Detection of Fe(III) ions Based on Solvents. Egytian J. Chem..

[18] Talbot D., Queiros Campos J., Checa-Fernandez B.L., Marins J.A., Lomenech C., Hurel C., Godeau G.D., Raboisson-Michel M., Verger-Dubois G., Obeid L. (2021). Adsorption of Organic Dyes on Magnetic Iron Oxide Nanoparticles. Part I: Mechanisms and Adsorption-Induced Nanoparticle Agglomeration. ACS Omega.

[19] Zygmunt Marczenko M.B. (2000). Separation, Preconcentration and Spectrophotometry in Inorganic Analysis.

[20] Taromi A.A., Kaliaguine S. (2018). Hydrodeoxygenation of triglycerides over reduced mesostructured Ni/γ- alumina catalysts prepared via one-pot sol-gel route for green diesel production. Appl. Catal..

[21] Rendo D. (2021). Adsorption of Methylene Blue Dye using Fe_3_O_4_ Magnetized Natural Zeolite Adsorbent. J. Kim. Sains Apl..

[22] Selvi P.P. (2019). Mass Transfer Enhancement for CO_2_ Absorption in Structured Packed Absorption Column. J. Chem. Soc. Pak..

[23] Asokan K. (2011). Mass Transfer Concepts.

[24] El-Sayed Y., Bandosz T.J. (2001). A Study of Acetaldehyde Adsorption on Activated Carbons. J. Colloid Interface Sci..

[25] Luz-Asunción M.D., Sánchez-Mendieta V., Martínez-Hernández A.L., Castaño V.M., Velasco-Santos C. (2015). Adsorption of phenol from aqueous solutions by carbon nanomaterials of one and two dimensions: Kinetic and equilibrium studies. J. Nanomater..

[26] Weber W.J., Morris J.C. (1963). Kinetics of Adsorption on Carbon from Solution. J. Sanit. Eng. Div..

[27] Banat F., Al-Bashir B., Al-Asheh S., Hayajneh O. (2000). Adsorption of Phenol by Bentonite. Environ. Pollut..

[28] El Maguana Y., Elhadiri N., Bouchdoug M., Benchanaa M., Jaouad A. (2019). Activated Carbon from Prickly Pear Seed Cake: Optimization of Preparation Conditions Using Experimental Design and Its Application in Dye Removal. Int. J. Chem. Eng..

[29] Medjor W., Wepuaka C., Godwill S. (2015). Spectrophotometric Determination of Phenol in Natural Waters by Trichloromethane Extraction Method after Steam Distillation. Int. Res. J. Pure Appl. Chem..

